# 

**Published:** 1897-04

**Authors:** 


### Communicated.

### DENTISTS IN THE U. S. ARMY.

*To the Dentists of the United States:*

At a recent meeting of the Ohio State Dental Society, a resolution was adopted advocating the employment of dentists in the United States Army. Said resolution briefly refers to the necessity for dental services among the enlisted men, recommending that Congress adopt such measures as will provide this special service, also recommending that every dental society in the land adopt a like course in furthering the interests of humanity.

The resolution also provided for the appointment of a Committee of one, who is empowered to represent the interests of the Ohio State Dental Society in this direction.

This Government expects its soldiers at all times to render prompt and efficient service and demands primarily that the recruit be physically sound. It has an established standard of soundness, and in order to prevent others than those possessing the requisite standard from being accepted, subjects each applicant to a medical examination which determines his fitness or unfitness to serve in the army of his country.

Furthermore, the Government not content alone with what the enlisted man *is* physically, attempts to maintain the standard and environs him with comfortable and sanitary quarters, furnishes him sufficient and wholesome food, exacts adequate and appropriate exercise and personal cleanliness. In short, throws about him every safeguard known to science for the perpetuation of his physical welfare.

Furthermore, should he be stricken by disease or disabled from any cause, he is cared for in modernly equipped hospitals and attended by skilled surgeons. Thus every need seems to be provided for.

But is this absolutely true? how about his dental organs? Surely the soldier is human; therefore his teeth are an important part of his physique and if they become diseased or are extracted, the whole organism suffers more or less as a consequence. It seems not a little strange that this Nation should not ere this, have included in its medical service the treatment of teeth.

Granted, that the entrance examination is thorough and includes in its requirements the possession of a sound set of teeth. There still remains the susceptibility to the insidious process of dental caries, which is no respector of persons and has no limit. Diseases may come and go, but dental caries when once started, like the brook "go on for ever."

The mere act of passing from civil into military life does not render a man immune from dental caries, and it is well known that men thus afflicted suffer much, indeed are often totally disabled for a greater or less period from discharging their duties. Therefore it seems only reasonable that some adequate provision be made to supply this branch of medical science, thereby improving and to that extent completing the medical service in the army.

Thus far, the only remedial process at the soldiers command is to have offending teeth extracted. This operation is usually performed by the hospital stewards, men without dental training, from whom it would be unreasonable to expect the necessary knowledge and skill to successfully practice this branch of surgery. Even if they were expert extractors of teeth, the immediate result of their operations would be the loss of what were intended for useful organs, the certain impairment of masticating facilities—and who can fortel the ultimate effects upon the whole constitution ?

Indifference to the needs of her servants is not in keeping with the generous policy which usually characterizes this Nation, therefore the dentists and medical men of this country, by virtue of their knowledge of existing conditions, earnestly appeal to every member of Congress to aid in the passage of a law, that will supply this long felt want.

Dentists, as members of the examining boards would undoubtedly be valuable acquisitions in preventing the admission of men with defective teeth, in this particular alone rendering valuable service. Furthermore, in cases of gunshot wounds and other injuries about the mouth and face, the dentists special knowledge of this region and his skill in devising and adapting appliances that may be necessary for their treatment, pre eminently qualify him to manage this class of cases, which the vicissitudes of war often present.

Altogether considering, the dentist is a necessary factor no less in the army than in civil communities.

Dear reader, will you not personally lend a helping hand in this noble cause? will you not enlist the services of your representative in Congress, to support an enactment with this end in view.

If this matter is brought to the minds of those who rule the affairs of our nation, we may hope that they will speedily add this branch of healing to their otherwise perfect system for the care of those who are the safeguard of frontiers and coast.

OTTO ARNOLD,
Representing the Ohio State Dental Society.

---

### ILLINOIS STATE DENTAL SOCIETY.

The thirty-third annual meeting of the Illinois State Dental Society will be held at Peoria, May 11 to 14, inclusive, 1897. An excellent program is in the course of preparation. Members are urgently requested to be present. The profession, generally, is cordially invited. A reduced rate of one fare and a third has been granted from all points within the State.

LOUIS OTTOFY, Sec'y,
Masonic Temple, Chicago.

### To Readers and Correspondents.

Communications intended for publication, and exchange journals and papers, should be addressed to the Editor of The American Journal of Dental Science, No. 845 N. Eutaw St. Baltimore, Md. All letters relating to the business of the Journal, or containing cash remittances, to be directed to Snowden & Cowman Mfg. Co., 9 W. Fayette Street, Baltimore, Md. Articles for publication should be received by the Editor at least two weeks previous to the first of the month on which the number in which it is designed for them to appear is issued.

☞ The American Journal of Dental Science will contain fifty pages of reading matter in each number, making a volume of about Six Hundred pages,

EXCLUSIVE OF ADVERTISEMENTS.

